# Modification of eating habits and lifestyle during COVID-19 in university students from Mexico and Peru

**DOI:** 10.3389/fnut.2024.1388459

**Published:** 2024-07-02

**Authors:** Claudia Milagros Arispe-Alburqueque, Fernando Luis Díaz del Olmo-Morey, César Arellano Sacramento, Benjamín Dario Sánchez-Mendoza, Martha Patricia López-González, Judith Soledad Yangali-Vicente, Miguel Ipanaqué-Zapata, Aldo Alvarez-Risco, Shyla Del-Aguila-Arcentales, Jaime A. Yáñez, Tania Ivette Alvarado-Santiago, Marx Engels Morales-Martínez

**Affiliations:** ^1^Universidad Tecnológica del Perú, Lima, Peru; ^2^Escuela de Posgrado, Universidad Privada Norbert Wiener, Lima, Peru; ^3^Universidad Estatal del Valle de Ecatepec, EDOMEX, Mexico; ^4^Facultad de Responsabilidad Social, Universidad Anahuac, Mexico City, Mexico; ^5^Universidad Privada Norbert Wiener, Vicerrectorado de Investigación, Lima, Peru; ^6^Universidad Tecnológica del Perú, Lima, Peru; ^7^Escuela de Posgrado, Universidad San Ignacio de Loyola, Lima, Peru; ^8^Facultad de Educación, Carrera de Educación y Gestión del Aprendizaje, Universidad Peruana de Ciencias Aplicadas, Lima, Peru; ^9^Universidad Nacional Federico Villarreal, Lima, Peru

**Keywords:** eating habits, lifestyle, nutrition, quality of life, physical activity, COVID-19, Peru, Mexico

## Abstract

**Objective:**

It was to evaluate changes in lifestyle habits and health behavior among university students in Peru and Mexico during periods of confinement associated with the COVID-19 pandemic and to identify possible relationships between these changes and sociodemographic variables, health status, and technology consumption.

**Methods:**

It was a quantitative, observational, and cross-sectional study conducted among a population of 739 Mexican students and 305 Peruvian students, most of whom were women (*n* =778, 74.5%) and non-graduates (*n* =921, 88.2%). The questionnaire scale for changes in lifestyles during the quarantine period has been previously validated.

**Results:**

The association between sociodemographic factors and dimensions of change in healthy lifestyles was evaluated, and it was shown that gender and country of residence were significant for all dimensions of healthy lifestyle (*p*  < 0.05), except for the level of education, which did not show significance about the change in the dimensions of media consumption (*p* = 0.875) and physical activity (*p* = 0.239). Within the dimensions mentioned, it can be stated that women are more likely than men to change their eating habits (adjusted prevalences (aPR) = 1.08, *p*  < 0.001), media consumption (aPR = 1.04, *p*  < 0.001), and physical activity (aPR = 1.02, *p*  < 0.001). Meanwhile, participants from Peru are more likely than participants from Mexico to change physical activity (aPR = 1.14, *p*  < 0.001) and media consumption (aPR = 1.22, *p*  < 0.001). Finally, graduate students were more likely than undergraduate students to change eating habits (aPR = 1.09, *p*  = 0.005) and unhealthy habits (aPR = 1.06, *p*  = 0.030).

**Conclusion:**

It was concluded that there were lifestyle changes in Mexican and Peruvian university students in their eating habits, physical activity, internet consumption, and food delivery.

## Introduction

The COVID-19 pandemic originated in Wuhan, China, and has become a major public health challenge worldwide (1, 2). The disease’s epicenter migrated from Europe to America, gradually expanding to all Latin American countries between February and March (3), including Mexico and Peru. In this sense, the governments implemented a series of measures to avoid further contagion, such as total and targeted quarantines, a reduction in the use of public transport, the temporary suspension of face-to-face work, and the change from the face-to-face educational model to an eminently virtual one to control the spread of the disease among more residents (4, 5).

The world’s population was urged to isolate themselves and refrain from social interaction due to the COVID-19 pandemic, which limited their ability to carry out their daily activities. Because of this, they have affected their lifestyles, reducing their physical activity by promoting a sedentary lifestyle and using YouTube videos to guide their exercises (6) and improving their physical activities.

Now, if healthy lifestyles are attitudes and behaviors that people adopt to prevent and improve their health, these have been affected by emotional aspects that people face in confinement (7). Regarding lifestyle guidelines, it has been recommended to maintain a healthy nutritional status and engage in physical exercise at home to manage the COVID-19 outbreak (8). Research on university students’ lifestyles in times of pandemic in various physical spaces has shown changes in sleep patterns, sexual activity, use of screens, food intake, or physical activity. However, research in two Latin American populations is insufficient (9). The study’s main objective was to evaluate changes in lifestyle habits and health behaviors among university students in Peru and Mexico during periods of confinement associated with the COVID-19 pandemic and identify possible relationships between these changes and sociodemographic variables, health status, and technology consumption.

## Materials and methods

### Study design

The research had a quantitative approach, a descriptive scope, and a non-experimental cross-sectional design. The population (*N* = 1044) consisted of undergraduate and graduate students from two universities in Mexico and Peru.

### Participants

The participants were Mexican and Peruvian undergraduate and graduate university students in the context of the pandemic from November 2021 to March 2022, under the international academic and research cooperation agreement between Norbert Wiener University and the State University of Ecatepec Valley. The sample was obtained through non-probability sampling for convenience at universities in Mexico and Peru, achieving a sample size of 1044 students. The inclusion criteria were being enrolled in the institutions at the time of the questionnaire; in the case of Norbert Wiener University, students from the Graduate School of the Master Program in Health Management participated; and in the case of the Universidad Estatal del Valle de Ecatepec, undergraduate students from the degrees of Gerontology and Medical Sciences participated. The reason for the inclusion of postgraduate students is that they constitute a set of sedimented knowledge that makes up the pool of previous experience that each of them has, and their participation is representative and very enriching. In the case of undergraduate students, they allowed us to guarantee representativeness, diversity, impact on educational policies, and opportunities for academic and professional training. Another requirement was that they must be over 18 years of age and answer at least 50% of the questionnaire provided. Given that all participants met the inclusion criteria and completed the entire questionnaire, the final sample size was 1044 students. The sample was obtained through non-probability sampling for convenience at universities in Mexico and Peru, achieving a sample size of 1044 students.

### Variables and instruments

The Vera-Ponce et al. (10) instrument was used with four dimensions: eating habits, harmful habits, physical activity, and use of media, with a total of 25 questions. Each question had four response options, each of which is equivalent to a score of no consumption = 1, decreased = 2, did not change = 3, increased = 4, which had a reliability of 0.80 through Cronbach’s alpha (10). The instrument can be observed in Appendix 1.

The cultural diversity between Peru and Mexico makes it possible to notice nominative and lexical differences in the denomination of the foods that the instrument used, for which it was necessary to carry out a cultural validation to translate from its original context to its Mexican equivalent, (1) explored the term (nominative word) in the Royal Spanish Academy Dictionary (11) and the Dictionary of Americanisms (12). With the definition obtained from each food, (2) the nutritional constitution was investigated in the Peruvian Tables of Food Composition (13) to contrast according to the term and characteristics with its equivalent in the Mexican System of Equivalent Foods (14), the Official Mexican Standard for essential health services. The criteria to provide guidance corroborated its characteristics with the Food Guides for the Peruvian Population (15). It was categorized as “1” if there was a change (values 2 and 4) and “0” if there was no change (values 1 and 3). In addition, a data Research Topic form was used for the sociodemographic variables of sex, country (Mexico–Peru), and level of study (undergraduate or postgraduate). Age is not included.

### Procedures

Due to the COVID-19 context and in-person restrictions, a virtual approach was implemented to invite student participation. With the necessary approvals in place, coordination was carried out with the study’s universities, wherein the teachers utilized the study chat platform as the primary channel to encourage students to complete the online questionnaire through Google Forms. Through this approach, we achieved active and diverse participation from both undergraduate and postgraduate students in our study.

### Statistical analysis

In the descriptive data analysis, measures of central tendency, including frequency and percentage, were employed. The statistical package, IBM SPSS Statistics 26.0, was utilized. In the inferential analysis, both the chi-square statistic and Fisher’s test were used with a significance level of 0.05, as appropriate. Subsequently, the association of sociodemographic factors (sex and educational level) with the dimensions of a healthy lifestyle was assessed, and finally, the associated factors (sex, educational level, cigarette consumption, alcohol consumption, physical activity, television consumption, radio consumption, and internet consumption) with changes in dietary habits were evaluated based on dimensions and the overall scale. For the assessment of association, Poisson regression with robust variance was employed, accounting for country-specific adjustments, including the standard error by country. Poisson regression with robust variance was used to model binary categorical variables and to avoid potential overdispersion of variance that may occur when modeling this type of variable. Two regression models were presented: the crude model assessed each independent variable against dependent variables, while the adjusted model incorporated the independent variables significant in the crude model (*p* < 0.05) along with confounding variables (country of residence, gender, and educational level). The primary statistical indicator for the first regression model was the crude prevalence (PR), and for the second model, adjusted prevalences (aPR) were used, both presented with 95% confidence intervals and *p*-values. The crude models for assessing the association of the associated factors with changes in eating habits were included in the Appendix 1. Additionally, before the association evaluations, the Joint F Test was conducted to verify that the levels of the factors against the dependent variables were significantly different (*p* < 0.05; Appendices 2, 3).

### Ethical aspects

The research has been approved by the institutional research ethics committee of Norbert Wiener Private University (exp. no. 1091-2021). Similarly, all the procedures dictated by the Declaration of Helsinki were carried out, such as the participants’ informed consent and the institutions’ authorizations.

## Results

The sociodemographic characteristics according to changes in lifestyle show that most students present lifestyle changes, with the main sociodemographic characteristic being more prominent in Peru (96.4%), in the female sex (92.9%), and in postgraduate students (97.6%). There is a significant difference between the categories of participants who presented lifestyle changes according to each sociodemographic characteristic (*p* < 0.05; Table 1).

**Table 1:** Sociodemographic characteristics according to lifestyle changes.

Lifestyle changes								
	No change		Yes, there was a change		Total	X^2^	Gl	*p*-value
Country	*N*	%	*N*	%				
Mexico	76	10.3	663	89.7	739	12.6	1	<0.001*
Peru	11	3.6	294	96.4	305			
**Sex**								
Male	32	3.08	234	22.41	266	6.4	1	0.014**
Female	55	5.26	723	69.25	778			
**Level of study**								
Postgraduate	3	2.4	120	97.6	123	6.3	1	0.008**
Undergraduate	84	9.1	837	90.9	921			
**Total**	**87**	**8.3**	**957**	**91.7**	**1044**			

*The test used was the chi-square test. **The test used was Fisher’s exact test.

**Figure 1:**
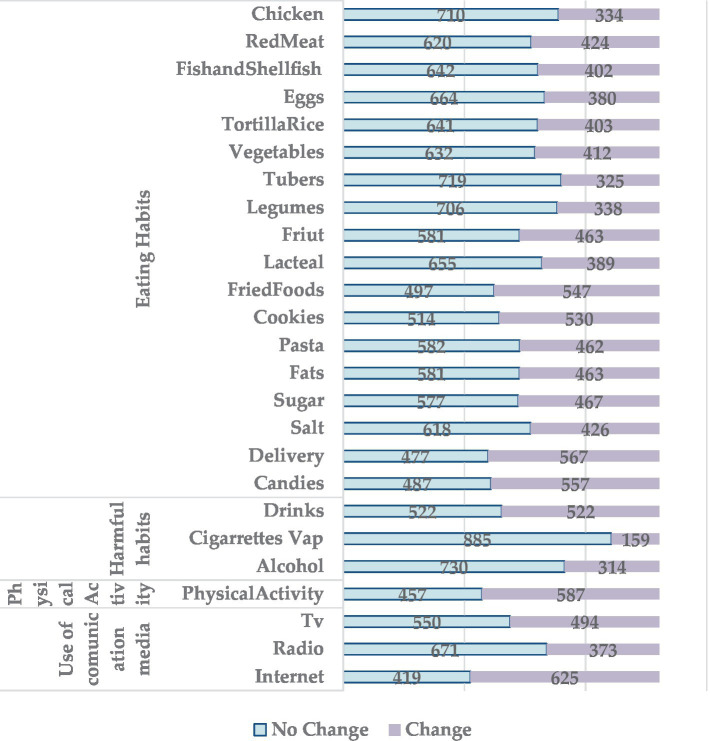
Frequency of changes in lifestyles during quarantine.

Figure 1 shows that in 19 of the 25 items of the instrument, “there was no change” in lifestyle habits. However, items such as “consumption of fried foods,” “consumption of bread and cookies,” “consumption of food by delivery,” and “consumption of sweets,” corresponding to the dimension of eating habits, exhibited a higher frequency in the category “there was a change,” indicating a decrease in these types of consumption. Similarly, “consumption of physical activity,” corresponding to the dimension of physical activity, and “internet consumption,” corresponding to the dimension of media usage, show a higher frequency in the category “there was change,” specifying that a majority displayed a decrease in physical activities and an increase in internet consumption.

With the association between sociodemographic factors and dimensions of change in healthy lifestyles, it is demonstrated that gender and country of residence are significant for all dimensions of healthy lifestyle (*p* < 0.05), except for educational level, which shows no significance concerning the change in media consumption (*p* = 0.875) and physical activity dimensions (*p* = 0.239). Within the mentioned dimensions, it can be asserted that women are more likely than men to change their eating habits (aPR = 1.08, *p* < 0.001), media consumption (aPR = 1.04, *p* < 0.001), and physical activity (aPR = 1.02, *p* < 0.001). Meanwhile, participants from Peru have a higher likelihood than participants from Mexico to change physical activity (aPR = 1.14, *p* < 0.001) and media consumption (aPR = 1.22, *p* < 0.001). Finally, postgraduate students were more likely than undergraduate students to change eating habits (aPR = 1.09, *p* = 0.005) and harmful habits (aPR = 1.06, *p* = 0.030; Tables 2–5).

**Table 2:** Association of sociodemographic factors with eating habits.

Variables	D1: change in eating habits					
	No change	Change				
	*n* (%)	*n* (%)	PR [95% CI]	*p*-value	aPR [95% CI]	*p*-value
**Sex**						
Man	44 (16.54%)	222 (83.46%)	Reference		Reference	
Woman	74 (9.51%)	704 (90.49%)	1.08 [1.05–1.11]	<0.001	1.08 [1.04–1.11]	<0.001
**Education level**						
Undergraduate	113 (12.27%)	808 (87.73%)	Reference		Reference	
Postgraduate	5 (4.07%)	118 (95.93%)	1.09 [1.02–1.16]	0.005	1.02 [1.01–1.05]	<0.001
**Country**						
Mexico	103 (13.94%)	636 (86.06%)	Reference		Reference	
Peru	15 (4.92%)	290 (95.08%)	1.10 [1.06–1.15]	<0.001	1.09 [1.06–1.11]	<0.001

Variables from the crude model with *p* < 0.05 are included in the adjusted model. The confounders of country, gender, and educational level are included in the adjusted model. PR: prevalence ratio, aPR: Adjusted prevalence ratio. ^a^In the adjusted model for ‘Change in the consumption of harmful habits’ only gender is significant, thus the adjusted model is not conducted.

**Table 3:** Association of sociodemographic factors with media consumption.

Variables	D2: change in media consumption^a^					
	No change	Change				
	*n* (%)	*n* (%)	PR [95% CI]	*p*-value	aPR [95% CI]	*p*-value
**Sex**						
Man	120 (45.11%)	146 (54.89%)	Reference		Reference	
Woman	337 (43.32%)	441 (56.68%)	1.05 [1.04–1.07]	<0.001	1.04 [1.03–1.07]	<0.001
**Education level**						
Undergraduate	408 (44.30%)	513 (55.70%)	Reference		Reference	
Postgraduate	49 (39.84%)	74 (60.16%)	1.17 [1.05–1.29]	0.002	1.01 [0.96–1.04]	0.875
**Country**						
Mexico	339 (45.87%)	400 (54.13%)	Reference		Reference	
Peru	118 (38.69%)	187 (61.31%)	1.22 [1.14–1.31]	<0.001	1.22 [1.20–1.24]	<0.001

Variables from the crude model with *p* < 0.05 were included in the adjusted model. The confounders of country, gender, and educational level were included in the adjusted model. PR: prevalence ratio, aPR: adjusted prevalence ratio.

a In the adjusted model for ‘Change in the consumption of harmful habits’ only gender is significant, thus the adjusted model is not conducted.

**Table 4:** Association of sociodemographic factors with harmful habits.

Variables	D3: change in the consumption of harmful habits					
	No change	Change				
	*n* (%)	*n* (%)	PR [95% CI]	*p*-value	aPR [95% CI]	*p*-value
**Sex**						
Man	159(59.77%)	107(40.23%)	Reference		Reference	
Woman	526(67.61%)	252(32.39%)	0.81[0.78–0.82]	<0.001	0.81 [0.79–0.82]	<0.001
**Education level**						
Undergraduate	606 (65.80%)	315 (34.20%)	Reference		Reference	
Postgraduate	79 (64.23%)	44 (35.77%)	1.04 [0.98–1.12]	0.170	1.06 [1.01–1.11]	0.030
**Country**						
Mexico	484 (65.49%)	255 (34.51%)	Reference		Reference	
Peru	201(65.90%)	104(34.10%)	0.95 [0.82–0.99]	0.043	0.97 [0.95–0.99]	0.008

Variables from the crude model with *p* < 0.05 were included in the adjusted model. The confounders of country, gender, and educational level were included in the adjusted model. PR: prevalence ratio, aPR: adjusted prevalence ratio. ^a^ In the adjusted model for “Change in the consumption of harmful habits” only gender is significant, thus the adjusted model is not conducted.

**Table 5:** Association of sociodemographic factors with physical activity.

Variables	D4: Change in physical activity					
	No change	Change				
	*n* (%)	*n* (%)	PR [95% CI]	*p*-value	aPR [95% CI]	*p*-value
**Sex**						
Man	120 (45.11%)	146 (54.89%)	Reference		Reference	
Woman	337 (43.32%)	441 (56.68%)	1.03 [1.02–1.05]	<0.001	1.02 [1.01–1.05]	<0.001
**Education level**						
Undergraduate	408 (44.30%)	513 (55.70%)	Reference		Reference	
Postgraduate	49 (39.84%)	74 (60.16%)	1.08 [1.02–1.13]	0.004	0.97 [0.93–1.02]	0.239
**Country**						
Mexico	339 (45.87%)	400 (54.13%)	Reference		Reference	
Peru	118 (38.69%)	187 (61.31%)	1.13 [1.01–1.26]	0.028	1.14 [1.12–1.16]	<0.001

Variables from the crude model with *p* < 0.05 were included in the adjusted model. The confounders of country, gender, and educational level were included in the adjusted model. PR: prevalence ratio, aPR: Adjusted prevalence ratio. ^a^In the adjusted model for “Change in the consumption of harmful habits” only gender is significant, thus the adjusted model is not conducted.

With respect to the factors associated with changes in eating habits, country, sex, education level, cigarette consumption, alcohol, physical activity, television, and internet use were reported (*p* < 0.005). The change in cigarette consumption (increase or decrease) presents a higher risk than people who do not change their cigarette consumption in presenting changes in eating habits (increase: aPR = 1.19, *p* < 0.001; decrease: aPR = 1.34, *p* < 0.001, respectively). Similarly, the change in alcohol consumption (increase or decrease) presents a higher risk of change in consumption of fast or processed food compared to people who do not change their alcohol consumption (increase: aPR = 1.27, *p* < 0.001, decrease: aPR = 1.26, *p* = 0.024). The change in physical activity (increase or decrease) presents a higher risk of change in the consumption of meat, chicken, and fish compared to people who do not change their physical activity intensity routine (Increase: aPR = 1.12, *p* = 0.002; Decrease: aPR = 1.28, *p* = 0.024). Finally, the decrease in television consumption (aPR = 1.13, *p* < 0.001) and increase in internet consumption (aPR = 1.11, *p* < 0.001) present a greater risk of changing eating habits compared to people who did not change their consumption routine (Table 6).

**Table 6:** Factors associated with eating habits according to dimensions and total scale.

	D1: change in the consumption of eggs, rice, vegetables, tubers, vegetables, tubers and dairy products		D2: change in consumption of bread and/or toast, noodles, margarine/butter, sugar and salt		D3: consumption of fried foods, fast food (delivery), sweets/desserts and sodas and/or processed beverages		D4: consumption of poultry, red meat and meat by-products, and fish and/or shellfish		Change in eating habits (Overall)	
Variables	aPR [95% CI]	*p*-value	aPR [95% CI]	*p*-value	aPR [95% CI]	*p*-value	aPR [95% CI]	*p*-value	aPR [95% CI]	*p*-value
**Country**										
Mexico	Reference		Reference		Reference		Reference		Reference	
Peru	1.09 [1.03–1.15]	0.002	1.02 [0.96–1.09]	0.537	1.06 [1.03–1.1]	0.001	1.16 [1.08–1.24]	<0.001	1.02 [0.98–1.07]	0.301
**Sex**										
Man	Reference		Reference		Reference		Reference		Reference	
Woman	1.07 [1.05–1.08]	<0.001	1.06 [1.03–1.09]	<0.001	0.99 [0.98–1]	0.117	1.22 [1.06–1.4]	0.004	1.04 [1.02–1.07]	<0.001
**Education level**										
Undergraduate	Reference		Reference		Reference		Reference		Reference	
Postgraduate	1.02 [1–1.05]	0.099	0.97 [0.95–0.99]	0.001	1 [0.99–1.02]	0.66	1.01 [1–1.01]	<0.001	1.01 [0.98–1.05]	0.402
**Cigarette consumption**										
No change	Reference		Reference		Reference		Reference		Reference	
No consumption	1.24 [1.16–1.32]	<0.001	1.59 [1.33–1.89]	<0.001	2.05 [1.79–2.35]	<0.001	1.43 [1.4–1.45]	<0.001	1.36 [1.32–1.41]	<0.001
Decreased	1.34 [1.21–1.47]	<0.001	1.75 [1.44–2.14]	<0.001	2.29 [1.96–2.66]	<0.001	1.25 [1.25–1.25]	<0.001	1.41 [1.39–1.42]	<0.001
Increased	1.19 [1.12–1.26]	<0.001	1.86 [1.58–2.19]	<0.001	2.1 [1.68–2.62]	<0.001	1.45 [1.35–1.55]	<0.001	1.35 [1.26–1.45]	<0.001
**Alcohol consumption**										
No change	Reference		Reference		Reference		Reference		Reference	
No consumption	1.12 [1.08–1.16]	<0.001	1.19 [1.18–1.2]	<0.001	1.19 [0.96–1.47]	0.116	1.2 [1.14–1.26]	<0.001	1.12 [1.09–1.16]	<0.001
Decreased	1.19 [1.03–1.37]	0.018	1.32 [1.25–1.39]	<0.001	1.26 [1.03–1.53]	0.024	1.31 [1.16–1.47]	<0.001	1.2 [1.12–1.29]	<0.001
Increased	1.24 [1.06–1.45]	0.008	1.17 [1.13–1.2]	<0.001	1.27 [1.13–1.42]	<0.001	1.42 [1.42–1.43]	<0.001	1.18 [1.16–1.2]	<0.001
**Physical activity**										
No change	Reference		Reference		Reference		Reference		Reference	
No consumption	1.15 [1.04–1.27]	0.008	1.11 [1.02–1.21]	0.018	1.04 [1.01–1.07]	0.006	1.19 [1.1–1.29]	<0.001	1.05 [1.01–1.09]	0.007
Decreased	1.16 [0.92–1.45]	0.2	1.19 [1.17–1.22]	<0.001	1.13 [1.02–1.25]	0.014	1.28 [1.03–1.59]	0.024	1.1 [1.07–1.13]	<0.001
Increased	1.11 [1–1.24]	0.054	1.13 [0.98–1.3]	0.105	0.97 [0.94–0.99]	0.017	1.12 [1.04–1.2]	0.002	1.04 [1.02–1.06]	<0.001
**Television consumption**										
No change	Reference		Reference		Reference		Reference		Reference	
No consumption	1.15 [1.14–1.17]	<0.001	1.1 [1.03–1.17]	0.003	1.02 [0.98–1.06]	0.306	1.2 [1.15–1.24]	<0.001	1.11 [1.06–1.16]	<0.001
Decreased	1.21 [1.19–1.24]	<0.001	1.21 [1.15–1.28]	<0.001	1.14 [1.1–1.18]	<0.001	1.25 [1.19–1.32]	<0.001	1.13 [1.11–1.16]	<0.001
Increased	1.1 [1.02–1.19]	0.011	1.11 [1.11–1.11]	<0.001	1.12 [1.11–1.14]	<0.001	1.17 [1.03–1.33]	0.018	1.11 [1.05–1.14]	<0.001
**Radio consumption**										
No change	Reference		Reference		Reference		Reference		Reference	
No consumption	1.09 [1.08–1.1]	<0.001	1.13 [1.13–1.14]	0	–	–	0.96 [0.96–0.97]	<0.001	1.06 [1.03–1.08]	<0.001
Decreased	1.14 [1.07–1.23]	<0.001	1.16 [1.1–1.22]	0	–	–	1.12 [1.09–1.15]	<0.001	1.07 [1–1.14]	0.052
Increased	1.02 [0.97–1.09]	0.408	1.15 [1.03–1.29]	0.013	–	–	1.12 [0.92–1.37]	0.251	1.04 [0.96–1.14]	0.304
**Internet use**										
No change	Reference		Reference		Reference		Reference		Reference	
No consumption	0.85 [0.85–0.86]	<0.001	0.77 [0.58–1.03]	0.081	0.76 [0.64–0.9]	0.002	0.78 [0.76–0.79]	<0.001	0.8 [0.77–0.83]	<0.001
Decreased	1.14 [1.14–1.14]	<0.001	0.96 [0.8–1.15]	0.644	1.03 [0.97–1.09]	0.364	1.19 [0.96–1.47]	0.116	0.99 [0.94–1.04]	0.602
Increased	1.38 [1.2–1.58]	<0.001	1.18 [1.01–1.39]	0.039	1.17 [1.03–1.33]	0.02	1.13 [0.95–1.35]	0.17	1.11 [1.05–1.17]	<0.001

^1^Variables from the crude model with *p* < 0.05 were included in the fitted model. The confounders of country, sex, and educational level were included in the adjusted model. PR: ratio prevalence, aPR: adjusted prevalence ratio. ^2^The crude prevalences are found in the Supplementary material.

## Discussion

In terms of relevant findings, the sample consists of 1044 students, mainly from Mexico (70.8%) and females (74.5%). Significant changes are observed in the lifestyles of women. The results indicate that, in general, 91.7% of the students show changes in lifestyles in Mexico and Peru, being significantly higher in Peru with 96.4% (*p* = 0.000). There is a significant and more remarkable change in postgraduate studies (97.6%), which agrees with Espinoza-Gutierrez et al. (16). It also coincides with Martínez (17), who found that graduate students in Colombia during the pandemic had a decrease in beverage consumption, increased physical activity, and did not add sugar or salt to their meals. In this case, it is important to note that the research is carried out with nutrition students. Graduate versus undergraduate college students have different ways of approaching stressful situations; this could be due to the difference in maturity and lifestyles (18).

Similarly, it is found that in the eating habits dimension (items 11, 12, 17, 18—consumption of fried foods, bread and/or cookies, take-out food, and consumption of sweets, respectively), in the physical activity dimension (item 22—physical activity) and the media use dimension (item 25—internet consumption), the alternative “there was a change” had a higher frequency. This agrees with Villaseñor et al. (19), who reported that during COVID-19 confinement, there was an increase in the consumption of unhealthy foods among Mexicans. The study by Murillo et al. (20), which included students from 10 countries, found a greater probability of following a prudent eating pattern when living in Mexico (OR:1.57) and Peru (OR:1.65).

The present study agrees with Bou-Hamad et al. (21), who found that approximately two-thirds (63.5%) of their participants adopted a healthy diet during the pandemic. Maté-Muñoz et al. (22) also mention a healthy change in eating habits compared to 12 months before the COVID-19 pandemic. Monteiro and Ferreira-Pêgo (23), when comparing eating habits during confinement compared to the normal semester of classes, found that the former was “better”; however, it is closely followed in percentage by the category being “the same.” On the other hand, Eşer et al. (24) indicate that they found changes in the order of meals in students during their distance education, with 31.7% who regularly consumed their main meals followed by 31.2% who jumped. Rafraf et al. (25), carried out only in women, when asking about the maintenance of the frequency of a regular eating pattern, report that eating habits changed during the pandemic, presenting both increases and decreases, only being stable in the face of the pandemic—item “Three to four times a week”.

It can be said from the literature that university students made a change for the better in their eating habits. This would be beneficial since it has been found that students who maintained a healthy diet during the pandemic indicated a better quality of life (21). During the pandemic, there was a transition from an institutional environment to a domestic one. This led the student to form new eating habits and a possibly healthier eating context. In this situation, the time dedicated to feeding (homemade) and the social part improved (26).

By relating the sex variable, we found that women had greater changes in their diet by 90.49% compared to authors such as Murillo et al. (20) and Miller et al. (27), who mention not finding a significant relationship. In contrast, Ferrara et al. (28) mention that being a woman was a predictor (OR:2.7) of a greater risk of unhealthy eating behaviors, both before and after the pandemic; and being older during the pandemic.

In addition, the diet presents alterations with a higher caloric intake of fats and carbohydrates, which has been reported as a coping mechanism to deal with elevated levels of anxiety and stress (26). This correlates with the social culture in Latin American countries such as Mexico and Peru, which are accustomed to greater closeness and physical contact, and during the pandemic social isolation generated a significant change with detrimental mental effects (29). Monteiro and Ferreira-Pêgo (23) found that the “consumption of fries and savories,” “consumption of juices and sugar-sweetened beverages,” and “consumption of alcoholic beverages” had a relevant increase between the normal period and the confinement period during the pandemic. An important and beneficial aspect is the information on how physical exercise was growing during the pandemic through digital platforms such as YouTube. In addition, the information conveyed in the videos was always guided by the importance of the global #stayathome campaign and looking for alternatives to perform the physical exercise routine in the home environment. This brings a new concept that should probably be applied during and after the COVID-19 pandemic: the so-called hybrid home-based physical exercise with virtual and online physical training guidance and prescription.

Regarding the dimension of physical activity, the alternative was greater (increase or decrease) and presented a greater risk of change in the consumption of meat, poultry, and fish compared to people who did not change the intensity of their physical activity routine. This correlates with the fact that public health decisions to prevent the spread of COVID-19 have led to the temporary closure of parks, gyms, and sports schools, causing a negative impact on the lifestyle of people and reducing the possibilities of physical activity and exercise (30). Regarding physical activity, studies such as Bou-Hamad et al. (21) and Eşer et al. (24) mention a negative change in exercise frequency during a pandemic. Monteiro and Ferreira-Pêgo (23) found an increase in physical exercise between the normal and confinement periods.

In addition the reduction in physical activity, there was an increase in internet consumption, which correlates with previous results that indicate that the use of digital technology has increased significantly worldwide (31). Likewise, Padilla (32) stated that schools and universities had to migrate to platforms to teach classes over the Internet through teleconferences and videoconferences, making Internet use indispensable during the pandemic. Internet users in Mexico reached 71.0% of the population during the pandemic, according to the National Institute of Statistics and Geography (33). In Peru, the increase in Internet use was accompanied by an increase in the use of media equipment (34). Bou-Hamad et al. (21) mention that the majority of the participants in their study (70%) used the Internet for at least three hours a day. Eşer et al. (24) found no difference in the time spent on social media and sex during the pandemic.

It is necessary to mention that in the harmful habits dimension, there were no changes in the consumption of cigarettes and alcohol (*n* = 885 and *n* = 730). However, the changes with frequencies of cigarette and alcohol consumption of *n* = 159 and *n* = 314, respectively (increase or decrease), were related to the change in cigarette consumption, which presents a greater risk of having changes in eating habits. Likewise, the change in alcohol consumption presents a greater risk of change in fast or processed food consumption. Bou-Hamad et al. (21) found that approximately 12% mentioned an increase in cigarette smoking and alcohol consumption during the pandemic. According to Zhang et al. (35), increased screen time, decreased physical activity, increased consumption of soft drinks and tea (also called consumption of sugar-sweetened beverages), use of alternative medicines or food supplements (including Chinese herbal medicines and vitamins), and less frequent meals were correlated with increased depression and anxiety.

Likewise, Espinoza-Gutierrez et al. (16), in a study carried out with students in Peru on eating habits and lifestyles, found that the perception of health was 95.49% satisfactory; only 4% rated it as low. The non-predominant healthy habits were not smoking and doing physical activity, while stress and not consuming healthy foods predominated unhealthy habits. While Ortiz et al. (36), in an investigation carried out in Chiapas, Mexico, with university students, observed an increase in the consumption of meals with an increase in processed meats, cookies or pastries, and sugary drinks, the consumption of fruits and vegetables decreased without this being significant. For Reyes Diaz et al. (37), in a study carried out with university students in Veracruz, Mexico, it was found that physical activity decreased, a fact that could be related to weight gain, while the consumption of fruits, vegetables, and legumes had ambivalent results.

The results agree with Balanzá-Martínez et al. (3), who reported that unhealthy lifestyles such as poor quality diet, physical inactivity, and tobacco and alcohol consumption are the main contributors to the global burden of disease. Similarly, Kim et al. (38) reported that one of the significant factors affecting preventive behaviors in college students related to COVID-19 was alcohol and tobacco use. Finally, our study showed that the impact of social isolation due to COVID-19 caused changes in the lifestyles of university students in the different dimensions studied, such as eating habits, harmful habits, physical activity, and media use.

Among the limitations that the study had were related to filling out the questionnaire in both countries since it was applied at a time when the academic cycles were closing. Another limitation is that the respondents were mostly from Mexico and female, which must be taken into account when generalizing the results; however, it is worth mentioning that the studies (18, 20–24, 26–28) had a higher frequency of female sex.

It must also be considered that the universities were public and private (this may represent economic differences). Likewise, it is not mentioned whether the students lived at home with their families or alone, as this may influence their habits; it is not mentioned whether the fast food or delivery was cheaper. The cross-sectional design was the only feasible approach owing to the retrospective self-report nature of changes during the pandemic. While a longitudinal methodology might have better established the temporal dynamics, the transversal study still provides valuable insights into the prevalence of reported modifications. One potential limitation is that only the sociodemographic variables of country, gender, and educational level were considered as potential confounding variables, based on previous evidence. However, it is plausible that other meaningful confounders that were not measured in this study could influence the association between the independent and dependent variables. Nevertheless, including these key variables in the adjusted model allowed for some control of confounding and yielded more accurate estimates of the associations under investigation. Other limitations to include may be the inability to establish causality and limitations in the generalizability of results. In addition, there may be potential biases associated with self-reported data and the conduct of online surveys, including social desirability bias and non-response bias.

Finally, one more limitation is that the direction of the change is not known; it does not change; that is, it is not known if habits or behaviors increase or decrease. Despite the limitations, it is considered that the study has strengths, one of which is a cross-cultural study carried out under conditions of confinement. In addition, despite being Latin countries and having the same language, a cultural validation was carried out for the name of the food, which gives solidity to the study and avoids confusion. The study also provides data from the region.

The study has several strengths: it presents information on the changes in the lifestyles of university students in two universities in the global south, Peru and Mexico, which provides the scientific literature with information on what happened in both places, caused by the COVID-19 pandemic. We account for the variations during the confinement period on changes in eating habits, harmful habits, consumption of toxic substances, physical activity, and use of the media, and the methodological tool used makes it possible to compare the results with other investigations that allow expanding the multiregional comparative mosaic.

However, there are some items that can enrich the tool we use to delve into some issues and shed light on explaining the phenomenon, and not just do it in descriptive terms. An example of this is knowing who the student lives with—family or fellow students. The approximate cost of meals prepared at home and purchased is delivery. We can find out that there was a change, but we do not have information on whether these changes are kept up-to-date and how they could be in other emerging scenarios.

In “local” terms, it gives us an account of the differences between two universities, one private and the other state, from two nations with similar economic dimensions but which have many differences internally, such as families that are financially allowed to pay a school fee and others enroll their children in public schools, which represents a lower financial outlay, but perhaps it is proportionally higher in terms of the family nucleus, elements that were not addressed in the study, but information between income and expenses can be interchanged to analyze the data on that slope.

In collegial terms, the study served to strengthen relationships between different academic groups into one, which makes it possible to interact with other researchers interested in learning more about Latin America or serves to consider a future cross-cultural analysis in relation to food, consumption of toxic substances, physical activity, etc. Despite everything that can be believed, there are similarities between Peruvians and Mexicans. Beyond the Spanish language, which is denoted in the food equivalents, the same food is named differently. The Latin American region has nuances in the nominal differences of the language; we highlight the cultural validation carried out of the foods that can be taken up in new investigations or between human groups from different regions.

We point out that the data presented in the study show a population that is mostly female, something that is possibly a Latin American phenomenon in which universities that offer undergraduate studies in the area of health are being occupied by women, possibly because they are associated with the Latin culture, care for the female sex, or possibly a generational cultural change, an element that will have to be explored and exposed in the future research as well.

## Conclusion

University students’ lifestyles changed during COVID-19 in Mexico and Peru regarding their eating habits, physical activity, internet consumption, and food delivery. In addition, postgraduate students from Peru had the highest frequency in the alternative where there was change. It is important to continue investigating the issue of changes in lifestyles in times not only of COVID-19 but in the face of any other contingency since it impacts university students; likewise, we must retake instruments that allow us to see the direction of the change and the dimension of mental health.

## Data availability statement

The original contributions presented in the study are included in the article/Supplementary material, further inquiries can be directed to the corresponding author.

## Ethics statement

The studies involving humans were approved by the research has been approved by the institutional research ethics committee of the Norbert Wiener Private University (exp. no. 1091-2021). The studies were conducted in accordance with the local legislation and institutional requirements. The participants provided their written informed consent to participate in this study.

## Author contributions

CA-A: Conceptualization, Data curation, Formal analysis, Investigation, Methodology, Software, Validation, Visualization, Writing – original draft, Writing – review & editing. FD: Conceptualization, Methodology, Software, Validation, Visualization, Writing – original draft, Writing – review & editing. CA: Conceptualization, Software, Supervision, Validation, Visualization, Writing – original draft, Writing – review & editing. BS: Data curation, Investigation, Software, Supervision, Validation, Visualization, Writing – original draft, Writing – review & editing. ML: Investigation, Methodology, Supervision, Validation, Visualization, Writing – original draft, Writing – review & editing. JY-V: Conceptualization, Data curation, Software, Validation, Visualization, Writing – original draft, Writing – review & editing. MI: Data curation, Formal analysis, Investigation, Supervision, Visualization, Writing – original draft, Writing – review & editing. AA: Data curation, Validation, Visualization, Writing – original draft, Writing – review & editing. SD: Data curation, Validation, Visualization, Writing – original draft, Writing – review & editing. JY: Data curation, Supervision, Visualization, Writing – original draft, Writing – review & editing. TA-S: Funding acquisition, Resources, Supervision, Visualization, Writing – original draft, Writing – review & editing. MM: Data curation, Funding acquisition, Resources, Validation, Visualization, Writing – original draft, Writing – review & editing.

## References

[1] Del-Aguila-Arcentales S Alvarez-Risco A Villalobos-Alvarez D Carhuapoma-Yance M Yáñez JA. COVID-19, mental health and its relationship with workplace accidents. Int J Ment Health Promot. (2022) 24:503–9. doi: 10.32604/ijmhp.2022.020513

[2] Yan J Kim S Zhang SX Foo M-D Alvarez-Risco A Del-Aguila-Arcentales S. Hospitality workers’ COVID-19 risk perception and depression: a contingent model based on transactional theory of stress model. Int J Hosp Manag. (2021) 95:102935. doi: 10.1016/j.ijhm.2021.102935, PMID: 36540684 PMC9756832

[3] Balanzá-Martínez V Atienza-Carbonell B Kapczinski F De Boni RB. Lifestyle behaviours during the COVID-19 – time to connect. Acta Psychiatr Scand. (2020) 141:399–400. doi: 10.1111/acps.13177, PMID: 32324252 PMC7264786

[4] Alvarez-Risco A Del-Aguila-Arcentales S Yáñez JA Rosen MA Mejia CR. Influence of technostress on academic performance of university medicine students in Peru during the COVID-19 pandemic. Sustain For. (2021) 13:8949. doi: 10.3390/su13168949

[5] Joo JY. Abrupt transition to remote learning in nursing students during the COVID-19 pandemic. J Nurs Educ. (2024) 63:108–15. doi: 10.3928/01484834-20231031-01, PMID: 37966424

[6] Vancini RL Viana RB dos Santos M Andrade CA de Lira B Nikolaidis PT. YouTube as a source of information about physical exercise during COVID-19 outbreak. Int J Sport Stud Health. (2022) 4:e123312. doi: 10.5812/intjssh.123312

[7] Ministerio de Salud del Perú. (2019). "Actividad física y estilos de vida saludables ayudan a prevenir más de 10 tipos de cáncer. Nota de Prensa [Physical activity and healthy lifestyles help prevent more than 10 types of cancer. Press release]." accessed 08/08/2022. Available at: https://www.gob.pe/institucion/minsa/noticias/52480-actividad-fisica-y-estilos-de-vida-saludables-ayudan-a-prevenir-mas-de-10-tipos-de-cancer.

[8] Ghram A Briki W Mansoor H Al-Mohannadi AS Lavie CJ Chamari K. Home-based exercise can be beneficial for counteracting sedentary behavior and physical inactivity during the COVID-19 pandemic in older adults. Postgrad Med. (2021) 133:469–80. doi: 10.1080/00325481.2020.1860394, PMID: 33275479

[9] Villavicencios V Guillermina N Merino EP Ramos FEE. Estilos de vida y calidad de vida en estudiantes universitarios en tiempo de Covid-19. Revista Universidad y Sociedad. (2020) 12:246–51.

[10] Vera-Ponce J Torres-Malca J Tello-Quispe E Orihuela-Manrique E De La Cruz-Vargas J. Validación de escala de cambios en los estilos de vida durante el periodo de cuarentena en una población de estudiantes universitarios de Lima, Perú [Validation of the scale of changes in lifestyles during the quarantine period in a population of university students in Lima, Peru]. Rev Fac Med Hum. (2020) 20:614–23. doi: 10.25176/RFMH.v20i4.3193

[11] Real Academia Española. (2014). "Diccionario de la lengua española (23a ed.) [Dictionary of the Spanish language (23rd ed.)]." accessed 08/08/2022. Available at: https://dle.rae.es.

[12] Asociación de Academias de la Lengua Española. (2010). "Diccionario de americanismos [Dictionary of Americanisms]." accessed 08/08/2022. Available at: https://asale.org/damer/

[13] Reyes García M., I. Gómez-Sánchez Prieto, and C. Espinoza Barrientos. (2017). "Tablas peruanas de composición de alimentos. Ministerio de Salud, Instituto Nacional de Salud [Peruvian food composition tables. Ministry of Health, National Institute of Health]." accessed 08/08/2022. Available at: https://repositorio.ins.gob.pe/xmlui/bitstream/handle/INS/1034/tablas-peruanas-QR.pdf?sequence=3&isAllowed=y.

[14] Pérez Lizaur A. González B. Palacios. (2014). "Sistema mexicano de alimentos equivalentes (4th ed.). Fomento de Nutrición y Salud, A. C. [Mexican System of Equivalent Foods (4th ed.). Promotion of Nutrition and Health, A.C.]." accessed 08/08/2022. Available at: https://fisiologia.facmed.unam.mx/wp-content/uploads/2019/02/2-Valoraci%C3%B3n-nutricional-Anexos.pdf.

[15] Ministerio de Salud del Perú. (2019). "Actividad física y estilos de vida saludables ayudan a prevenir más de 10 tipos de cáncer [Physical activity and healthy lifestyles help prevent more than 10 types of cancer]." accessed 08/08/2022. Available at: https://www.gob.pe/institucion/minsa/noticias/52480-actividad-fisica-y-estilos-de-vida-saludables-ayudan-a-prevenir-mas-de-10-tipos-de-cancer.

[16] Espinoza-Gutierrez GA Yance-Cacñahuaray G Runzer-Colmenares FM. Hábitos alimentarios y estilos de vida de los estudiantes de medicina a inicios de la pandemia Covid-19. Revista de la Facultad de Medicina Humana. (2022) 22:319–26. doi: 10.25176/RFMH.v22i2.4381

[17] Martínez K. Hábitos alimenticios y estilos de Vida en estudiantes del último año de la Carrera de nutrición y dietética de la Pontificia Universidad Javeriana [eating habits and lifestyles in students of the last year of the nutrition and dietetics career at the Pontificia Universidad Javeriana]. Trabajo de Grado: Pontificia Universidad Javeriana (2021).

[18] Zhao Y Ding Y Shen Y Failing S Hwang J. Different coping patterns among US graduate and undergraduate students during COVID-19 pandemic: a machine learning approach. Int J Environ Res Public Health. (2022) 19:2430. doi: 10.3390/ijerph19042430, PMID: 35206617 PMC8878508

[19] Lopez V Karen AM Garduño J Regules AEO Romero LMI Martinez OAG. Cambios en el estilo de vida y nutrición durante el confinamiento por SARS-CoV-2 (COVID-19) en México: un estudio observacional. Revista Española de Nutrición Humana y Dietética. (2021) 25:e1099. doi: 10.14306/renhyd.25.S2.1099

[20] Murillo AG Gómez G Durán-Agüero S Parra-Soto SL Araneda J Morales G. Dietary patterns and dietary recommendations achievement from Latin American college students during the COVID-19 pandemic lockdown. Front Sustain Food Syst. (2022) 6:299. doi: 10.3389/fsufs.2022.836299

[21] Bou-Hamad I Hoteit R Hijazi S Ayna D Romani M El Morr C. Coping with the COVID-19 pandemic: a cross-sectional study to investigate how mental health, lifestyle, and socio-demographic factors shape students’ quality of life. PLoS One. (2023) 18:e0288358. doi: 10.1371/journal.pone.0288358, PMID: 37471388 PMC10358926

[22] Maté-Muñoz JL Hernández-Lougedo J Ruiz-Tovar J Olivares-Llorente R García-Fernández P Zapata I. Physical activity levels, eating habits, and well-being measures in students of healthcare degrees in the second year of the COVID-19 pandemic. Healthcare. (2023) 11:1570. doi: 10.3390/healthcare11111570, PMID: 37297711 PMC10252304

[23] Monteiro M Ferreira-Pêgo C. University students eating habits: Normal semester vs. lockdown period caused by COVID-19 pandemic. Int J Environ Res Public Health. (2022) 19:12750. doi: 10.3390/ijerph191912750, PMID: 36232047 PMC9565997

[24] Durmaz E Sevinç AK Tunçer E. Effect of emotional eating and social media on nutritional behavior and obesity in university students who were receiving distance education due to the COVID-19 pandemic. J Public Health. (2023) 31:1645–54. doi: 10.1007/s10389-022-01735-x, PMID: 35891803 PMC9305038

[25] Rafraf M Molani-Gol R Sahebjam M. Effect of COVID-19 pandemic on eating habits and lifestyle of college students in Tabriz, Iran: a cross-sectional study. Front Public Health. (2023) 11:1185681. doi: 10.3389/fpubh.2023.1185681, PMID: 37601215 PMC10437127

[26] Hurtado HV Largacha V S Guerrero PI Galvez E P. Ambientes y hábitos alimentarios: Un estudio cualitativo sobre cambios producidos durante la pandemia por Covid-19 en estudiantes universitarios. Revista chilena de nutrición. (2022) 49:79–88. doi: 10.4067/S0717-75182022000100079

[27] Miller L Déchelotte P Ladner J Tavolacci M-P. Effect of the COVID-19 pandemic on healthy components of diet and factors associated with Unfavorable changes among university students in France. Nutrients. (2022) 14:3862. doi: 10.3390/nu14183862, PMID: 36145238 PMC9506412

[28] Ferrara M Langiano E Falese L Diotaiuti P Cortis C De Vito E. Changes in physical activity levels and eating behaviours during the COVID-19 pandemic: sociodemographic analysis in university students. Int J Environ Res Public Health. (2022) 19:5550. doi: 10.3390/ijerph19095550 35564943 PMC9105810

[29] Li Q Yang X Wang X Zhang H Ding N Zhao W. COVID-19 symptoms, internet information seeking, and stigma influence post-lockdown health anxiety. Front Psychol. (2023) 14:1228294. doi: 10.3389/fpsyg.2023.1228294, PMID: 37637921 PMC10448810

[30] Hurtado V Felipe A Ramos OA Jácome SJ del Mar M Cabrera M. Actividad física y ejercicio en tiempos de COVID-19. CES Medicina. (2020) 34:51–8. doi: 10.21615/cesmedicina.34.COVID-19.6

[31] Tala Á Vásquez E Plaza C. Estilos de vida saludables: una ampliación de la mirada y su potencial en el marco de la pandemia. Rev Med Chile. (2020) 148:1189–94. doi: 10.4067/S0034-98872020000801189, PMID: 33399785

[32] Padilla JJ. Análisis del comportamiento del tráfico en Internet durante la pandemia del Covid-19: el caso de Colombia. Entre Ciencia e Ingeniería. (2020) 14:26–33. doi: 10.31908/19098367.2012

[33] National Institute of Statistics and Geography. (2022). "Instituto Nacional de Estadística y Geografía [National Institute of Statistic and Geography] ", accessed 09/08/2022. Available at: https://www.inegi.org.mx/app/indicadores/?ind=6206972692&tm=6#D6206972692#D6206972693

[34] Contreras A Darío R Rafaele M de la Cruz G Álvarez LM Rodríguez SLQ. Impacto del aislamiento social por COVID-19 en los hábitos de consumo de los medios de comunicación en Perú. Revista Cubana de Información en Ciencias de la Salud. (2021) 32

[35] Zhang Y Tao S Yang Q Mou X Gan H Zhou P. Lifestyle behaviors and mental health during the coronavirus disease 2019 pandemic among college students: a web-based study. BMC Public Health. (2022) 22:2140. doi: 10.1186/s12889-022-14598-4, PMID: 36414957 PMC9682808

[36] Ortiz N Carlos J Fuentevilla GC. Alimentación y estilos de vida durante el confinamiento por pandemia en estudiantes universitarios de Chiapas, México. RESPYN. (2023) 22:29–37. doi: 10.29105/respyn22.1-709

[37] Reyes Diaz RA Yépez Arias KR Cruz Lara NM Sánchez RR Morales Barradas N Fonseca León E. Covid-19 y su impacto en hábitos de consumo alimentario en estudiantes universitarios del estado de Veracruz. Ciencia Latina Revista Científica Multidisciplinar. (2023) 7:5509–21. doi: 10.37811/cl_rcm.v7i1.4843

[38] Kim K-A Hyun MS De Gagne JC Ahn J-A. A cross-sectional study of nursing students' eHealth literacy and COVID-19 preventive behaviours. Nurs Open. (2023) 10:544–51. doi: 10.1002/nop2.1320, PMID: 36631729 PMC9537965

