# Supplementary material for: Modification of eating habits and lifestyle during COVID-19 in university students from Mexico and Peru
Source: Front Nutr. 2024 Jul 2;11:1388459. doi: 10.3389/fnut.2024.1388459 (PMC11250360; doi:10.3389/fnut.2024.1388459)
Supplement: Supplementary file 1 [file Table_1.DOCX]

Appendix 1. Factors associated with eating habits according to dimensions and total scale (crude model).

|  | **D1: Change in the consumption of eggs, rice, vegetables, tubers, vegetables, tubers and dairy products** | | **D2: Change in consumption of bread and/or toast, noodles, margarine/butter, sugar and salt** | | **D3: Consumption of fried foods, fast food (delivery), sweets/desserts and sodas and/or processed beverages** | | **D4: Consumption of poultry, red meat and meat by-products and fish and/or shellfish** | | **Change in eating habits (Overall)** | |  |
| --- | --- | --- | --- | --- | --- | --- | --- | --- | --- | --- | --- |
|  |  |  |  |  |  |  |  |  |  |  |  |
|  |  |  |  |  |  |  |  |  |  |  |  |
|  |  |  |  |  |  |  |  |  |  |  |  |
| **Variables** | **PR [95%IC]** | **p** | **PR [95%IC]** | **p** | **PR [95%IC]** | **p** | **PR [95%IC]** | **p** | **PR [95%IC]** | **p** |  |
| **Sex** |  |  |  |  |  |  |  |  |  |  |  |
| Man | Reference |  | Reference |  | Reference |  | Reference |  | Reference |  |  |
| Woman | 1.11[1.1-1.12] | <0.001 | 1.11[1.09-1.12] | <0.001 | 1.06[1.02-1.10] | 0.006 | 1.3[1.15-1.46] | <0.001 | 1.08[1.05-1.11] | <0.001 |  |
| **Education level** |  |  |  |  |  |  |  |  |  |  |  |
| Undergraduate | Reference |  | Reference |  | Reference |  | Reference |  | Reference |  |  |
| Postgraduate | 1.18[1.04-1.34] | 0.009 | 1.07[1.03-1.12] | 0.001 | 1.17[1.08-1.28] | <0.001 | 1.21[1.04-1.41] | 0.015 | 1.09[1.03-1.16] | 0.005 |  |
| **Cigarette consumption** |  |  |  |  |  |  |  |  |  |  |  |
| No change | Reference |  | Reference |  | Reference |  | Reference |  | Reference |  |  |
| No consumption | 1.78[1.54-2.05] | <0.001 | 2.24[1.85-2.71] | <0.001 | 2.59[1.95-3.45] | <0.001 | 2.09[1.99-2.2] | <0.001 | 1.69[1.53-1.86] | <0.001 |  |
| Decreased | 1.96[1.43-2.68] | <0.001 | 2.55[2.00-3.26] | <0.001 | 2.93[2.03-4.22] | <0.001 | 2.04[1.64-2.54] | <0.001 | 1.77[1.56-2.01] | <0.001 |  |
| Increased | 1.83[1.46-2.3] | <0.001 | 2.61[1.99-3.42] | <0.001 | 2.76[1.85-4.10] | <0.001 | 2.13[1.97-2.3] | <0.001 | 1.71[1.41-2.08] | <0.001 |  |
| **Alcohol consumption** |  |  |  |  |  |  |  |  |  |  |  |
| No change | Reference |  | Reference |  | Reference |  | Reference |  | Reference |  |  |
| No consumption | 1.46[1.22-1.75] | <0.001 | 1.62[1.38-1.90] | <0.001 | 1.66[1.13-2.43] | 0.009 | 1.69[1.47-1.94] | <0.001 | 1.4[1.23-1.59] | <0.001 |  |
| Decreased | 1.55[1.15-2.08] | 0.004 | 1.79[1.63-1.97] | <0.001 | 1.77[1.24-2.53] | 0.002 | 1.79[1.38-2.32] | <0.001 | 1.47[1.26-1.71] | <0.001 |  |
| Increased | 1.63[1.18-2.25] | 0.003 | 1.69[1.39-2.05] | <0.001 | 1.77[1.28-2.45] | 0.001 | 1.87[1.69-2.06] | <0.001 | 1.45[1.25-1.67] | <0.001 |  |
| **Physical activity** |  |  |  |  |  |  |  |  |  |  |  |
| No change | Reference |  | Reference |  | Reference |  | Reference |  | Reference |  |  |
| No consumption | 1.32[1.10-1.58] | 0.002 | 1.34[1.24-1.45] | <0.001 | 1.24[1.06-1.46] | 0.008 | 1.43[1.18-1.74] | <0.001 | 1.2[1.11-1.29] | <0.001 |  |
| Decreased | 1.43[1.06-1.93] | 0.02 | 1.53[1.33-1.76] | <0.001 | 1.45[1.15-1.84] | 0.002 | 1.63[1.16-2.27] | 0.004 | 1.3[1.16-1.47] | <0.001 |  |
| Increased | 1.43[1.17-1.75] | <0.001 | 1.49[1.47-1.51] | <0.001 | 1.3[1.15-1.47] | <0.001 | 1.41[1.25-1.6] | <0.001 | 1.26[1.15-1.37] | <0.001 |  |
| **Television Consumption** |  |  |  |  |  |  |  |  |  |  |  |
| No change | Reference |  | Reference |  | Reference |  | Reference |  | Reference |  |  |
| No consumption | 1.29[1.09-1.52] | 0.004 | 1.29[1.10-1.51] | 0.001 | 1.16[0.88-1.53] | 0.286 | 1.31[1.14-1.5] | <0.001 | 1.22[1.02-1.47] | 0.032 |  |
| Decreased | 1.49[1.28-1.73] | <0.001 | 1.51[1.34-1.70] | <0.001 | 1.38[1.07-1.79] | 0.014 | 1.59[1.37-1.84] | <0.001 | 1.3[1.11-1.53] | 0.001 |  |
| Increased | 1.46[1.24-1.72] | <0.001 | 1.45[1.12-1.89] | 0.005 | 1.45[1.13-1.84] | 0.003 | 1.54[1.38-1.71] | <0.001 | 1.3[1.07-1.58] | 0.009 |  |
| **Radio consumption** |  |  |  |  |  |  |  |  |  |  |  |
| No change | Reference |  | Reference |  | Reference |  | Reference |  | Reference |  |  |
| No consumption | 1.28[1.02-1.62] | 0.036 | 1.39[1.10-1.75] | 0.006 | 1.3[0.86-1.96] | 0.216 | 1.18[0.86-1.61] | 0.307 | 1.22[0.99-1.51] | 0.065 |  |
| Decreased | 1.43[1.09-1.87] | 0.009 | 1.51[1.29-1.77] | <0.001 | 1.42[0.99-2.05] | 0.059 | 1.51[1.12-2.05] | 0.007 | 1.28[1.03-1.58] | 0.027 |  |
| Increased | 1.42[1.09-1.85] | 0.01 | 1.56[1.13-2.14] | 0.007 | 1.4[0.96-2.02] | 0.077 | 1.56[1.06-2.3] | 0.025 | 1.3[1.02-1.66] | 0.036 |  |
| **Internet use** |  |  |  |  |  |  |  |  |  |  |  |
| No change | Reference |  | Reference |  | Reference |  | Reference |  | Reference |  |  |
| No consumption | 1.16[1.15-1.17] | <0.001 | 1.08[0.90-1.29] | 0.434 | 1[0.84-1.18] | 0.976 | 1.06[1-1.12] | 0.054 | 1.01[0.99-1.03] | 0.189 |  |
| Decreased | 1.59[1.50-1.68] | <0.001 | 1.44[1.31-1.58] | <0.001 | 1.45[1.26-1.67] | <0.001 | 1.73[1.49-2.02] | <0.001 | 1.27[1.18-1.37] | <0.001 |  |
| Increased | 1.61[1.46-1.77] | <0.001 | 1.46[1.27-1.67] | <0.001 | 1.47[1.33-1.64] | <0.001 | 1.47[1.41-1.53] | <0.001 | 1.29[1.22-1.36] | <0.001 |  |

The models were pairwise evaluated for each of the factors against each of the eating habits, taking into account country variance.

Appendix 2. Joint F-test to assess the levels of the independent variables in healthy lifestyle changes.

| **Variables** | **D1: Change in eating habits** | | **D2: Change in media consumption^a^** | | **D3: Change in the consumption of harmful habits** | | **D4: Change in physical activity** | |
| --- | --- | --- | --- | --- | --- | --- | --- | --- |
|  | **F-test** | **p** | **F-test** | **p** | **F-test** | **p** | **F-test** | **p** |
| **Sex** | **32.95** | <0.001 | 5626.96 | <0.001 | 222.41 | <0.001 | 76.28 | <0.001 |
| **Education level** | **8.05** | 0.0046 | 9.63 | 0.0019 | 9.75 | 0.032 | 8.21 | 0.0042 |

Appendix 3. Joint F-test to assess the levels of the independent variables in healthy eating

|  | **D1: Change in the consumption of eggs, rice, vegetables, tubers, vegetables, tubers and dairy products** | | **D2: Change in consumption of bread and/or toast, noodles, margarine/butter, sugar and salt** | | **D3: Consumption of fried foods, fast food (delivery), sweets/desserts and sodas and/or processed beverages** | | **D4: Consumption of poultry, red meat and meat by-products and fish and/or shellfish** | | **Change in eating habits (Overall)** | |  |
| --- | --- | --- | --- | --- | --- | --- | --- | --- | --- | --- | --- |
|  |  |  |  |  |  |  |  |  |  |  |  |
|  |  |  |  |  |  |  |  |  |  |  |  |
|  |  |  |  |  |  |  |  |  |  |  |  |
| **Variables** | **F-test** | **p** | **F-test** | **p** | **F-test** | **p** | **F-test** | **p** | **F-test** | **p** |  |
| **Sex** | 762.58 | <0.001 | 150.62 | <0.001 | 7.58 | 0.006 | 18.21 | <0.001 | 32.95 | <0.001 |  |
| **Education level** | 6.84 | 0.009 | 10.36 | 0.002 | 13.57 | 0.0002 | 5.93 | 0.015 | 8.05 | 0.005 |  |
| **Cigarette consumption** | 17.48 | <0.001 | 48.42 | <0.001 | 25.1 | <0.001 | 40.23 | <0.001 | 29.63 | <0.001 |  |
| **Alcohol consumption** | 8.91 | 0.003 | 28.76 | <0.001 | 6.74 | 0.009 | 19.24 | <0.001 | 24.36 | <0.001 |  |
| **Physical activity** | 5.4 | 0.0201 | 34.72 | <0.001 | 9.55 | 0.002 | 8.13 | 0.004 | 18.80 | <0.001 |  |
| **Television Consumption** | 8.52 | 0.0035 | 7.74 | 0.006 | 6.45 | 0.009 | 38.32 | <0.001 | 6.88 | 0.009 |  |
| **Radio consumption** | 6.78 | 0.0092 | 7.28 | 0.007 | 6.34 | 0.013 | 5.04 | 0.0248 | 4.4 | 0.036 |  |
| **Internet use** | 96.68 | <0.001 | 34.67 | <0.001 | 48.36 | 0.045 | 50.19 | <0.001 | 43.35 | <0.001 |  |
